# Can we safely reduce the administration of 131-iodine in patients with differentiated thyroid cancer? – experience of the Brugmann hospital in Brussels

**DOI:** 10.1186/s13044-020-00089-4

**Published:** 2020-09-12

**Authors:** Laura Iconaru, Felicia Baleanu, Georgiana Taujan, Ruth Duttmann, Linda Spinato, Rafik Karmali, Pierre Bergmann, Anne-Sophie Hambye

**Affiliations:** 1 Department of Endocrinology, CHU Brugmann, Université Libre de Bruxelles, Place van Gehuchten4, 1020 Laeken, Brussels, Belgium; 2 Department of Anatomopathology, CHU Brugmann, Université Libre de Bruxelles, Brussels, Belgium; 3 Department of Otorhinolaryngology, CHU Brugmann, Université Libre de Bruxelles, Brussels, Belgium; 4 Department of Nuclear Medicine, CHU Brugmann, Université Libre de Bruxelles, Brussels, Belgium

**Keywords:** Differentiated thyroid cancer, 131-iodine therapy, Therapeutic response

## Abstract

**Background:**

131-iodine (^131^I) administration after surgery remains a standard practice in differentiated thyroid cancer (DTC). In 2014, the American Thyroid Association presented new guidelines for the staging and management of DTC, including no systematic ^131^I in patients at low-risk of recurrence and a reduced ^131^I activity in intermediate risk.

The present study aims at evaluating the rate of response to treatment following this new therapeutic management compared to our previous treatment strategy in patients with DTC of different risks of recurrence.

**Methods:**

Patients treated and followed up for DTC according to the 2014-ATA guidelines (Group 2) were compared to those treated between 2007 and 2014 (Group 1) in terms of general characteristics, risk of recurrence (based on the 2015-ATA recommendations), preparation to ^131^I administration, cumulative administered ^131^I activity and response to treatment.

**Results:**

In total, 136 patients were included: 78 in Group 1 and 58 in Group 2. The two groups were not statistically different in terms of clinical characteristics nor risk stratification: 42.3% in Group 1 and 31% in Group 2 were classified as low risk, 38.5 and 48.3% as intermediate risk and 19.2 and 20.7% as high risk (*P* = 0.38). Two patients (one in each group) with distant metastases were excluded from the analysis.

Preparation to ^131^I administration consisted in rhTSH stimulation in 23.4% of the patients in Group 1 and 100% in Group 2 (*p* < 0.001).

^131^I was administered to 46/77 patients (59.7%) in Group 1 (5 at low risk of recurrence) and 38/57 patients (66.7%) in Group 2 (0 with a low risk). Among the patients treated by ^131^I, median cumulative activity was significantly higher in Group 1 (3.70GBq [100 mCi] range 1.11–11.1 GBq [30–300 mCi]) than in Group 2 (1.11 GBq [30 mCi], range 1.11–7.4 GBq [30–200 mCi], *P* < 0.001). Complete response was found in 90.9% in Group 1 vs. 96.5% in Group 2 (*P* = 0.20).

**Conclusions:**

Using the 2015-ATA evidence-based guidelines for the management of DTC, meaning no ^131^I administration in low-risk patients, a low activity in intermediate and even high risk patients, and a systematic use of rhTSH stimulation before ^131^I therapy allowed us to reduce significantly the median administered ^131^I activity, with a similar rate of complete therapeutic response.

## Background

Differentiated thyroid cancer (DTC), which includes papillary and follicular cancers, comprises the vast majority (> 90%) of all thyroid cancers [1]. Although its incidence has increased over the last few decades, DTC remains a rare malignant disease with a usually good prognosis.

Radioactive 131-iodine (RAI) ablation of residual thyroid tissue after thyroidectomy remains the cornerstone of post-surgical treatment for patients with DTC. The main goals of this treatment are to improve overall and disease-specific survival, to reduce the risk of persistent/recurrent disease and associated morbidity, and to facilitate follow-up. It is generally assumed that the first RAI administration after thyroidectomy is mainly aimed at destroying residual, presumably benign, remnant thyroid tissue, but also at treating suspected but undetected or unknown regional or distant metastases [2].

In DTC, maybe more than in other, more aggressive malignancies, minimizing treatment-related morbidity and side effects, and avoiding unnecessary therapy, are however issues that need to be taken into account in the therapeutic management due to the usually slow natural evolution of the disease. Because of the potential risks related to the exposure to ionizing radiation, selecting the appropriate patients as well as the optimal therapeutic protocol for successful treatment remains a challenge, requiring accurate disease staging and risk stratification [3].

In 2014, the American Thyroid Association (ATA) presented evidence-based guidelines for the staging and management of DTC. These guidelines consist in a dynamic risk assessment system, which classifies the patient based on the response to initial therapy (complete vs indeterminate, biochemical incomplete or structural incomplete response), and include the possibility of avoiding systematic complementary RAI therapy in low-risk patients and the use of recombinant human thyrotropin (rhTSH) preparation instead of thyroid hormone withdrawal for low- and intermediate-risk patients. rhTSH is currently approved by many international authorities to prepare the patient for RAI remnant ablation after near-total or total thyroidectomy for DTC without evidence of distant metastases, with a significantly better quality of life compared to the hormone withdrawal regimen [1, 2]. In 2015, we endorsed and followed the ATA-Guidelines for DTC for all our patients.

The primary aim of the current study was to evaluate the efficacy of low 131-iodine (^131^I) activity (including no administration in low risk patients) and of the systematic use of rhTSH before RAI on the rate of response to treatment or recurrence, compared to higher activities and hormonal withdrawal. As secondary aims, we analyzed the influence of this modified therapeutic management on the duration of hospital stay and reported treatment tolerance.

## Materials and methods

From 2015 on, all patients diagnosed and treated at our institution with histological confirmation of DTC have been prospectively enrolled. For the purpose of the current study, only patients treated until December 2017 were included, so that at least 2 years follow-up data were available. The study population (Group 2) was compared to of the patients treated between January 2007 and December 2014, used as historical control group (Group1). Within each group, patients were further subclassified based on the risk of structural disease recurrence as defined by 2015-ATA guidelines [1, 4]:

- Subgroup A (low-risk patients): intrathyroidal DTC, pT1a, clinical N0 or ≤ 5 pathologic N1 micrometastases (< 0.2 cm in largest dimension), M0;
- Subgroup B (intermediate-risk patients): pT1b, pT2, aggressive histology, minor extrathyroidal extension, vascular invasion, or > 5 involved lymph nodes (0.2–3 cm), M0;
- Subgroup C (high-risk patients): pT3, pT4, gross extrathyroidal extension, incomplete tumor resection, distant metastases, or lymph node > 3 cm. For the purpose of the present study, patients with distant metastases were excluded from the analysis.

In order to allow a correct comparison between both groups, all pathology data from the patients in Group 1 were reviewed by the same pathologist and reclassified according to the 2015-ATA classification.

All patients underwent total or near-total thyroidectomy. Peroperative exploration of lymph nodes was systematically performed, followed by lymph node dissection when appropriate. Therapeutic central-compartment neck dissection for patients with clinically involved central nodes accompanied total thyroidectomy. Prophylactic central-compartment neck dissection was considered in patients with clinically uninvolved central neck lymph nodes who have advanced primary tumors (T3 or T4) or clinically involved lateral neck nodes. Therapeutic lateral neck compartmental lymph node dissection was performed for patients with biopsy-proven metastatic lateral cervical lymphadenopathy.

Until 2014, decision to treat patients with DTC as well as the preparation regimen and the administered ^131^I activity were based on the 2009 ATA recommendations [5]. rhTSH was available since 2011, and used in preparation to RAI in low, and to a lesser degree, in intermediate-risk patients. From 2015 on, treatment management was based on the new ATA-recommendations, including no complementary ^131^I administration in low-risk patients, and an almost systematic use of rhTSH to prepare the patient for RAI remnant ablation even in high-risk patients.

All patients treated by ^131^I were hospitalized in a shielded room. During their hospital stay, they were visited at least twice daily, always by the same nuclear medicine physician. The emitted radiation was measured at 1 m of the patient’s chest wall during each visit using a dedicated portable dose rate meter (Radiagem™, Canberra Inc., France) calibrated according to the manufacturer’s specification and to which a 1 m-long cord was attached. Discharge was allowed when the measured dose-rate of radiation had decreased to a level defined by law (< 20 μSv/h at 1 m of the chest wall).

All patients received levothyroxine to maintain TSH at a level depending on the risk of recurrence and on clinical parameters.

Initial evaluation of postoperative disease status included measurement of serum thyroglobulin (Tg) and anti-Thyroglobulin antibody (AntiTgAb), neck ultrasonography (US), and post-therapy whole-body scintigraphy (WBS) for the patients treated with ^131^I.

Patients were followed up every 6 months from the time of initial treatment until 2019. Follow-up included a clinical examination, Tg and AntiTgAb dosage, as well as neck ultrasonography with 12–5 Mhz linear transducer. Tg and AntiTgAb dosage was performed by electrochemiluminescence test “ECLIA” using a Cobas e 801 immunoassay system; the accuracy was determined using Elecsys reagents, samples and controls according to the EP05 - A3 protocol from the CLSI (Clinical and Laboratory Standards Institute; reference values for Tg = 3.5–77 ng/mL with a limit of detection = 0.04 ng/mL, measuring range for AntiTgAb = 10–4000 IU/mL, with a limit of detection = 10 IU/mL).

In some patients with positive AntiTgAb in whom Tg measurement was less reliable, a diagnostic ^123^I-WBS after rh-TSH injection was realized.

At each visit, response to therapy was evaluated as complete or incomplete based on the 2015-ATA guidelines:

- complete response: no clinical, biochemical, or structural evidence of disease defined as a a Tg value of < 0.2 ng/mL under thyroid hormone replacement therapy, or of < 1 ng/mL after rhTSH stimulation, in the absence of structural or functional evidence of disease (and in the absence of AntiTgAb)
- biochemical incomplete response: abnormal suppressed Tg (≥1 ng/mL) and/or stimulated Tg values (≥10 ng/mL) or rising AntiTgAb with negative imaging
- structural incomplete response: persistent or newly identified loco-regional or distant metastases on US and/or WBS), independently of Tg and AntiTgAb
- indeterminate response: nonspecific biochemical or structural findings that cannot be confidently classified as either benign or malignant. This includes patients with stable or declining anti-Tg antibody levels without definitive structural evidence of disease, non-stimulated detectable Tg 0.2–1 ng/mL, stimulated Tg between 1 and 10 ng/mL, or AntiTgAb stable or declining in the absence of structural or functional disease.

The two groups were compared in terms of general characteristics, risk of recurrence (based on the 2015-ATA recommendations), cumulative administered ^131^I activity, preparation regimen for RAI therapy (thyroid hormone withdrawal versus rhTSH stimulation) and response to therapy. Duration of hospital stay and reported tolerance to RAI treatment were also compared. For the purpose of the current study, only patients with persistent evidence of complete response until 2019 were considered as in complete remission.

This study was approved by the local ethics committees (the Hospital Ethics Committee of CHU Brugmann, protocol number B077201836052).

### Statistical analyses

All statistical analyses were performed using IBM SPSS statistics. Qualitative variables were assessed with Chi-square test and numerical variables with parametric and non-parametric tests according to their distribution which was evaluated by the Kolmogorov-Smirnov test.

Mean and standard deviations, median and ranges were calculated as usual.

A *p* value of < 0.05 was considered as significant.

## Results

Hundred and thirty-six patients were included: 78 in Group 1 (63 women / 15 men, mean ± SD age at diagnosis: 50.3 ± 14.25 years) and 58 in Group 2 (42 women / 16 men, mean ± SD age at diagnosis: 46.8 ± 15.6 years).

The two groups did not differ significantly in terms of clinical, pathological and ATA-risk of recurrence (Table 1). In Group 1, 33 patients (42.3%) were classified as low risk (subgroup A), 30 (38.5%) as intermediate risk (subgroup B) and 15 (19.2%) as high risk (subgroup C). In group 2, there were respectively 18 patients (31%) in subgroup A, 28 (48.3%) in subgroup B and 12 (20.7%) in subgroup C (*P* = 0.38). Distant metastases were found in 2 patients (one in each group), who were excluded from the analysis.

**Table 1:** Patients’ characteristics at inclusion

Parameters	Group 1 (%)	Group 2 (%)	*P* value
Number of patients	78 (57.4%)	58 (42.6%)	
Gender			
- female	63 (80.8%)	42 (72.4%)	0.30
- male	15 (19,2%)	16 (27.6%)	
Mean ± SD age at diagnosis (years)	50.3 ± 14.25	46.8 ± 15.6	0.42
Histology			
- papillary	62 (79.5%)	44 (75.9%)	0.29
- papillary follicular variant	6 (7.7%)	6 (10.3%)	
- follicular	10 (12.8%)	8 (13.8%)	
TNM classification			
- I	58 (74.4%)	48 (82.7%)	0.38
- II	4 (5.1%)	5 (8.6%)	
- III	11 (19.0%)	4 (6.9%)	
- IVa	4 (6.9%)	0	
- IVb	0	0	
- IVc^a^	1 (1.3%)	1 (1.7%)	
ATA-recurrence risk stratification^b^			
- low-risk	33 (42.3%)	18 (31.0%)	0.38
- intermediate-risk	30 (38.5%)	28 (48.3%)	
- high-risk	15 (19.2%)	12 (20.7%)	
Median (range) duration of hospital stay (days)	3 (2–10)	1.5 (1–3)	< 0.001
Side effects (n)	Constipation (10)	0	< 0.001
	Headache (2)		
	Cervical pain (3)		
Response to the therapy			
- complete response	70 (90.9%)	55 (96.5%)	0.20
- biochemical incomplete response	2 (2.6%)	2 (3.5%)	
- structural incomplete response	3 (3.9%)	0	
- indeterminate response	2 (2.6%)	0	

^a^ patients with distant metastases were excluded for the analysis of the results

^b^**low-risk**: intrathyroidal DTC, pT1a, clinical N0 or ≤ 5 pathologic N1 micrometastases,M0; **intermediate-risk**: pT1b, pT2, aggressive histology, minor extrathyroidal extension, vascular invasion, or > 5 involved lymph nodes (0.2–3 cm), M0; **high-risk:** pT3, pT4, gross extrathyroidal extension, incomplete tumor resection, distant metastases, or lymph node > 3 cm,

In Group 1, RAI was administered to 5/33 patients (15.1%) in subgroup A and 27/30 patients (90%) in subgroup B. In Group 2, no patient in subgroup A received ^131^I therapy. In subgroup B, 27/28 (96.4%) patients were treated, and 1 patient refused RAI. All high-risk patients were treated in both groups.

After exclusion of the 2 metastatic patients, median cumulative activity of ^131^I calculated for the 77 patients in Group 1 was significantly higher (1.85 GBq, [50 mCi], range 0–11.1 GBq, [0–300 mCi]) than for the 57 patients in Group 2 (1.11 GBq, [30 mCi], range 0–7.4 GBq, [0–200 mCi], *P* = 0.01).

In both groups, the median cumulative activity included a large number of low-risk and some intermediate-risk patients who did not receive RAI, potentially not reflecting the true difference in administered activity between both groups. Therefore, we also calculated the median cumulative activity only in those patients who had effectively received ^131^I. The difference between both groups was even more striking: 3.7 GBq [100 mCi] (range 1.11–11.1 GBq [30–300 mCi]) for the 46 patients in Group 1, and 1.11 GBq [30 mCi] (range 1.11–7.4 GBq [30–200 mCi]) for the 38 patients in Group 2 (*p* < 0.001). Details regarding ^131^I activity according to the risk classification in the patients who indeed received RAI are reported in Table 2.

**Table 2:** Cumulative activity of ^131^I (in GBq [mCi]) depending on the risk classification for the non metastatic patients treated by RAI before 2015 (Group 1) and since 2015 (Group 2)

	Group 1			Group 2			*P* value
	n	Median	Range	n	Median	Range	
All risks	46	3.70 [100]	1.11–11.1 [30–300]	38	1.11 [30]	1.11–7.4 [30–200]	< 0.001
low-risk	5	3.70 [100]	1.11–3.70 [30–100]	0	N/A	N/A	< 0.001
intermediate-risk	27	3.70 [100]	1.11–11.1 [30–300]	27	1.11 [30]	N/A	< 0.001
high-risk^a^	14	5.55 [150]	1.85–9.25 [50–250]	11	2.67 [72]	1.11–7.4 [30–200]	< 0.001

*N/A* not applicable, *mCi* millicurie, *GBq* gigabecquerel

^a^ patients with distant metastases are excluded

In preparation to RAI therapy, rhTSH was used in 11/46 patients (23.9%) in Group 1 (3/5 with low risk, 7/27 with intermediate risk and 1/14 with high risk) and in all patients in Group 2 (27 intermediate risk and 11 high risk, *p* < 0.001). Treatment tolerance was excellent in all of them, with no related side effects. In contrast, mild to moderate side effects were reported in 15/46 (32.6%) patients in Group 1, all of them treated after thyroid hormone withdrawal (constipation (10), headache (2) and cervical discomfort/pain (3), this latter probably due to radiation-induced thyroiditis).

Duration of hospital stay after administration of ^131^I was significantly shorter in Group 2 (median (range): 1.5 (1-3) days versus (3 (2-10) days in Group 1, *p* < 0.001).

At follow-up, complete response was documented in 70/77 patients (90.9%) in Group 1 and in 55/57 (96.5%) in Group 2 (*p* = 0.20). Seven patients in Group 1 (9.1%) had an incomplete response in Group 1 (2 biochemical incomplete, 3 structural incomplete and 2 indeterminate response) and 2 (3.5%) in Group 2 (2 biochemical incomplete). The rate of complete response for patients at intermediate and high risk of recurrence prepared with rhTSH was similar in both groups (*p* = 0.42).

During follow-up, no DTC-related death was mourned. In Group 1, 2 patients (1 intermediate- and 1 high-risk) died from biopsy-proven other (lung, colorectal) malignancies.

## Discussion

In patients with DTC, ^131^I ablation of remnant functional thyroid tissue following (near) total thyroidectomy aims at reducing the risk of recurrence and/or mortality and at facilitating patients’ follow-up. However, because of the good prognosis of most DTC and of the possible risks associated with exposure to ionizing radiation, uncertainty has raised about the necessity of treating all DTC patients with ^131^I, as well as about the optimal amount of radioiodine required for effective ablation.

To address these issues, the American Thyroid Association (ATA) presented management guidelines in 2014, proposing a therapeutic approach for DTC in which postoperative staging based on pathology is recommended not only for assessing risk for recurrence and mortality, but also for tailoring decisions regarding the need for adjuvant therapy [1, 2, 4]. The guidelines also put forward the use of rhTSH preparation instead of thyroid hormone withdrawal for low- and intermediate-risk patients.

Since the publication of these guidelines, a standardized therapeutic protocol is applied to all patients without known metastases treated for DTC in our institution, consisting in no administration of ^131^I after surgery in low-risk patients and a low activity even in high-risk patients as well as the systematic use of rhTSH before iodine treatment. The primary goal of the present study was to evaluate the effect of this management strategy for DTC on the rate of response to treatment and/or recurrence compared to the previous treatment policy. Furthermore, because local toxicity of RAI and duration of hospital stay are related to the amount of administered activity, and the treatment tolerance is influenced by the preparation regimen, our secondary goal was to analyze the effect of the changes in management strategy on these parameters.

Data in the literature are somehow contradictory with regard to the efficacy of low dose RAI after surgery in patients with DTC. A systematic review of randomized and observational studies published in 2007 was inconclusive as to whether low-dose ^131^I (1.1GBq [30 mCi]) was associated with rates of ablation success similar to - or lower than - rates obtained with high-dose radioiodine (3.7 GBq [100 mCi]) [6]. More recently, other researchers reported that high dose of ^131^I resulted in a successful ablation more often than low dose [3, 7, 8], especially in those patients with positive anti thyroglobulin antibodies or higher stimulated thyroglobulin levels at the moment of radioiodine ablation [8].

In our patients, the rate of complete response since 2015 did not differ significantly from the historical control group regardless of the risk stratification patients, despite a significant decrease in ^131^I activity and no RAI in low-risk patients. The same rate of complete response was achieved by administering 1.1GBq [30 mCi] instead of 3.7 GBq [100 mCi] in patients with DTC. These observations are in agreement with other studies, which used the ablation success rate as a surrogate clinical end point for response to ^131^I. Castagna et al. provided the first evidence that in DTC patients, high RAI activities at ablation had no major advantage over low activities [9]. This was confirmed in a meta-analysis by Cheng et al. and large multicenter randomized trials [6, 10–15], showing that low-dose radioiodine (1.1GBq [30 mCi]) is as effective as a high-dose (3.7 GBq [100 mCi]) in ablating thyroid remnant in patients with DTC.

The use of low RAI activity has several advantages for the patient as well as for the community. First, patients have fewer side effects, both immediate and delayed, including a reduced risk of developing another primary cancer due to the exposure to ionizing radiations [11].

Second, reducing the RAI activity is cost-effective. Shortening the duration of hospital stay, and even avoiding hospitalization in case of low-risk DTC, significantly reduces the financial costs incurred by the health service provider. In our study, median hospitalization was 3 days before 2015 and 1.5 days after. Third, the duration of compulsory isolation, homestay and social distancing after hospital discharge is also reduced, allowing patients to resume work and normal life more rapidly. Lastly, administration of a lower activity decreases the radioactive waste and exposure to the environment [11].

2015 was a key year for the management of DTC patients in our institution: besides the adoption of a therapeutic approach based on the risk of recurrence, preparation to RAI by rhTSH administration became the standard of care regardless of the risk assessment. Since 2015, 100% of the patients have received rhTSH (vs 23.9% before), with a very similar high rate of complete therapeutic response even in the patients at a high risk of recurrence. As expected, tolerance to treatment improved noticeably, with no reported side effects after rhTSH injection, vs in 1/3 of the patients after thyroid hormone withdrawal, mostly related to hypothyroidism.

These observations confirm a recently published consensus paper stating that, in patients with ATA low- and intermediate-risks DTC without extensive lymph node involvement, and sometimes even in high-risk patients, preparation for RAI with rhTSH stimulation is an acceptable alternative to thyroid hormone withdrawal for achieving remnant ablation, based on evidence of superior short-term quality of life and noninferiority of remnant ablation efficacy [2].

The results of our study seem promising and may have important practical implications with regard to improvement of treatment, by making therapies safer and more convenient, with a higher quality of life. They sustain the personalized approach to treatment, follow-up and prognosis proposed by 2015 ATA guidelines, and are in line with the results of a recent meta-analysis by Vardarli et al. who showed for the first time that at the longer term, at least 2-year follow-up, recurrence rate among patients who had ^131^I ablation with 1.1 GBq was not higher than with 3.7 GBq [16].

However, the potential clinical impact of our results is currently limited by the relatively small number of patients already included, the use of historical controls, with potential biases related to differences in surgical techniques or recommended standards of care, as well as by the chosen minimal duration of follow-up of 2 years. This duration is an intermediate period compared with other studies, maybe too short given the known slow progression of DTC, yet also very recently used by Vardarli [16]. New patients continue to be included and more follow-up data will be available for analysis in the future.

## Conclusion

Compared to the administration of high ^131^I activities after thyroid hormone withdrawal for post-surgical ablation of remnant thyroid tissue in patients with non metastatic DTC, applying the 2015-ATA management guidelines, including no ^131^I administration in low-risk patients and rhTSH stimulation as a preparation regimen before RAI, allowed to achieve a very similar high rate of complete therapeutic response with significantly less administered ^131^I, a shorter hospital stay and an improved quality of life at less costs for the community.

## Data Availability

The datasets used and/or analysed during the current study are available from the corresponding author on reasonable request.

## Abbreviations

AntiTgAb: Anti-Thyroglobulin antibody
ATA: American Thyroid Association
DTC: Differentiated thyroid cancer
GBq: Gigabecquerel
mCi: Millicurie
RAI: Radioactive 131-iodine
rhTSH: Recombinant human thyrotropin
Tg: Thyroglobulin
US: Ultrasonography
WBS: Whole-body scintigraphy
